# The Occult Insulinoma Was Localized Using Endoscopic Ultrasound Guidance: A Case Report

**DOI:** 10.1002/ccr3.9634

**Published:** 2025-02-10

**Authors:** Zhongqiu Guo, Yanrong Chen, Ronghuo Liu, Yuhua Chen

**Affiliations:** ^1^ Department of Endocrinology and Metabolism, Longgang District People's Hospital of Shenzhen The Second Affiliated Hospital of the Chinese University of Hong Kong, Longgang Central City Shenzhen Guangdong People's Republic of China

**Keywords:** endoscopic ultrasound, fine needle aspiration biopsy technique, hypoglycemia, insulinoma

## Abstract

Insulinomas are the primary etiology of endogenous hyperinsulinemic hypoglycemia, which often manifest with Whipple’s triad and neuroglycopenic symptoms. Given the diverse clinical manifestation and subtle onset of insulinomas generally in a small size, detecting a minority of these generally small tumors can be challenging. We reported a case of a 44‐year‐old female patient with recurrent hypoglycemia accompanied by hyperinsulinemia, and the conventional imaging revealed no abnormality. With the aid of endoscopic ultrasound‐guided fine‐needle aspiration biopsy (EUS‐FNAB), the insulinoma was precisely diagnosed and localized, and successfully excised via operation. The patient’s hyperinsulinemia and hypoglycemic episodes were relieved significantly after surgery. The application of EUS‐FNAB notably enhances the diagnostic accuracy for occult insulinomas, thereby informing appropriate surgical management. Herein, we advocate for invasive EUS examination in patients exhibiting strong clinical and laboratory indicators of insulinoma, even when conventional imaging results are negative.

**Summary**

- The case report aim to deepen primary care clinicians' understanding of insulinoma.
- We advocate for invasive EUS examination in patients exhibiting strong clinical and laboratory indicators of insulinoma, even when conventional imaging results are negative.

## 1 Introduction

Insulinoma, a prevalent functional pancreatic neuroendocrine neoplasm (pNEN), is characterized by specific insulin secretion, leading to endogenous hyperinsulinemia and subsequently resulting in hypoglycemia [1]. Insulinoma is the predominant cause of hypoglycemia related to endogenous hyperinsulinemia, which occurs in approximately 1–4 individuals per million [2]. Recurrent hypoglycemia is a typical clinical manifestation of insulinoma, with about 92% of patients exhibiting the classic Whipple's triad. Other clinical manifestations also include psychiatric abnormalities, and neurological symptoms such as impaired consciousness and seizures [3, 4]. While qualitative diagnosis of insulinoma is straightforward, the challenge lies in localizing the often small and hidden tumor. Herein, in this report, we present a case of insulinoma with an occult tumor and provide a comprehensive review of the relevant literature, aiming to raise awareness among clinicians regarding early diagnosis and treatment of insulinoma.

## 2 Case History and Examination

A 44‐year‐old female patient was admitted to our department, following recurrent syncope and profuse sweating over the past 2 years, with worsening symptoms in the last month. This symptom tends to diminish following a meal, occurring roughly once every 6 months. The patient denied any speech abnormalities or hemiplegia. Previously, the patient received treatment at a local hospital, where an intravenous random blood glucose measurement revealed a level of 54.7 mg/dL (3.0 mmol/L), indicating hypoglycemia. Imaging studies, including chest, abdominal, and cranial CT scans, revealed no abnormalities, and thus no additional treatment was administered.

The patient started undergoing frequent seizures approximately 1 month ago, occurring once a week. Accompanying these seizures, she experienced impaired consciousness and incoherent speech, with symptom relief after eating. The patient was admitted to the hospital for evaluation of syncope. Over the past 2 years, the patient's dietary intake had increased, resulting in a weight gain of approximately 5 kg. Her medical history included a cesarean‐section hemorrhage, followed by regular menstruation after delivery. For the past 5 years, she had been dealing with hypertension, although her treatment adherence was inconsistent. There were no notable findings in her family medical history.

The patient's vital signs were stable during the physical examination. Her height measured 153 cm, weight 74 kg, with a BMI of 31.6 kg/m^2^. The patient was alert and provided relevant answers. No signs of acanthosis nigricans or hyperpigmentation were observed on the skin or mucous membranes. Axillary and pubic hair appeared within normal limits. No enlargement of the thyroid gland was noted. Cardiopulmonary and abdominal examinations revealed no abnormalities. Muscle strength and tone in the extremities were within normal limits. No pathological signs were detected.

## 3 Investigations and Diagnosis

**FIGURE 1:**
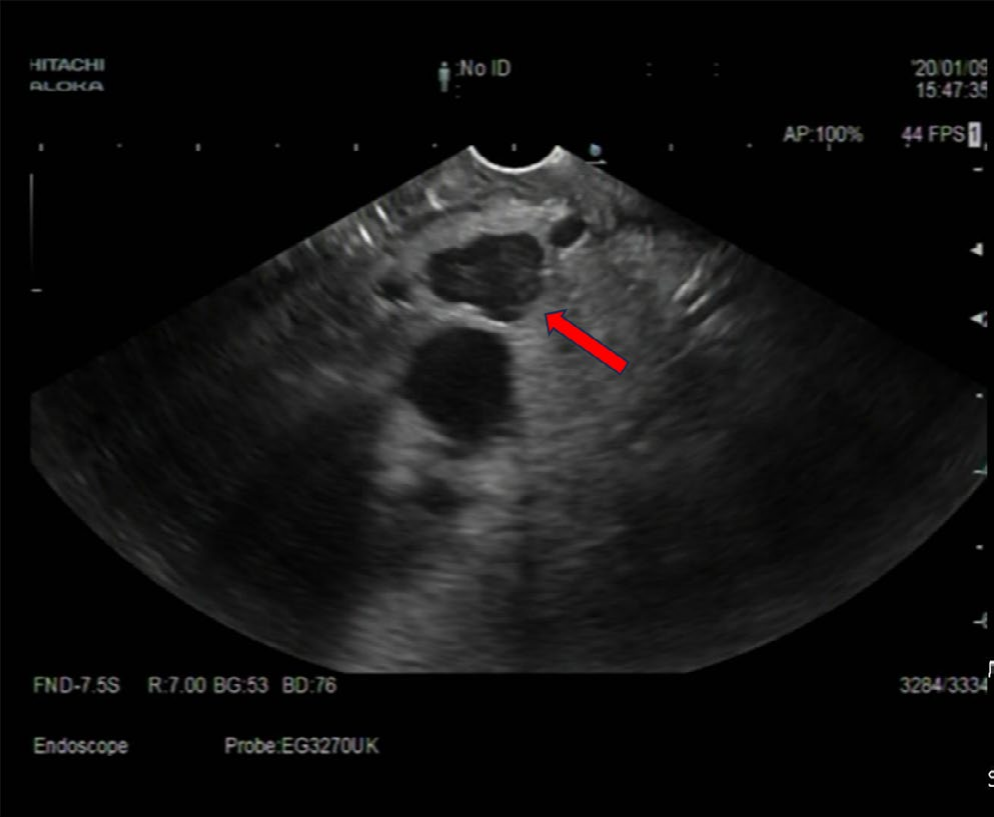
Tumor detected by endoscopic ultrasound.

**FIGURE 2:**
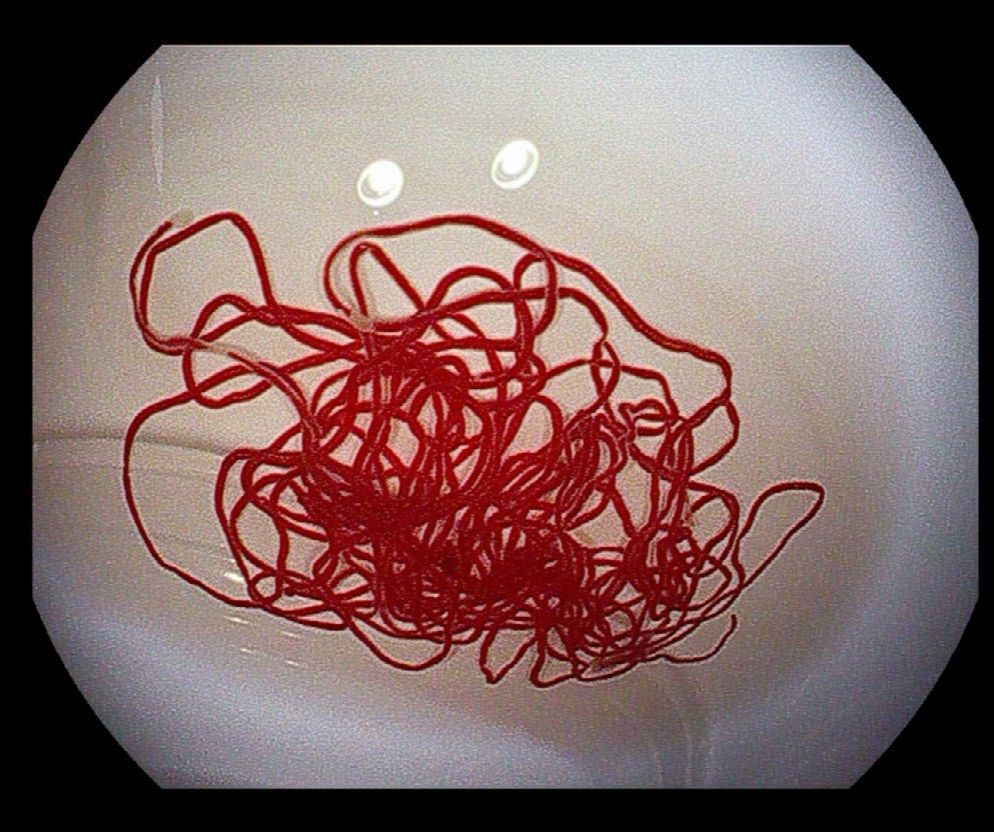
Strips of tissue visible by ultrasonic endoscopic fine‐needle.

**FIGURE 3:**
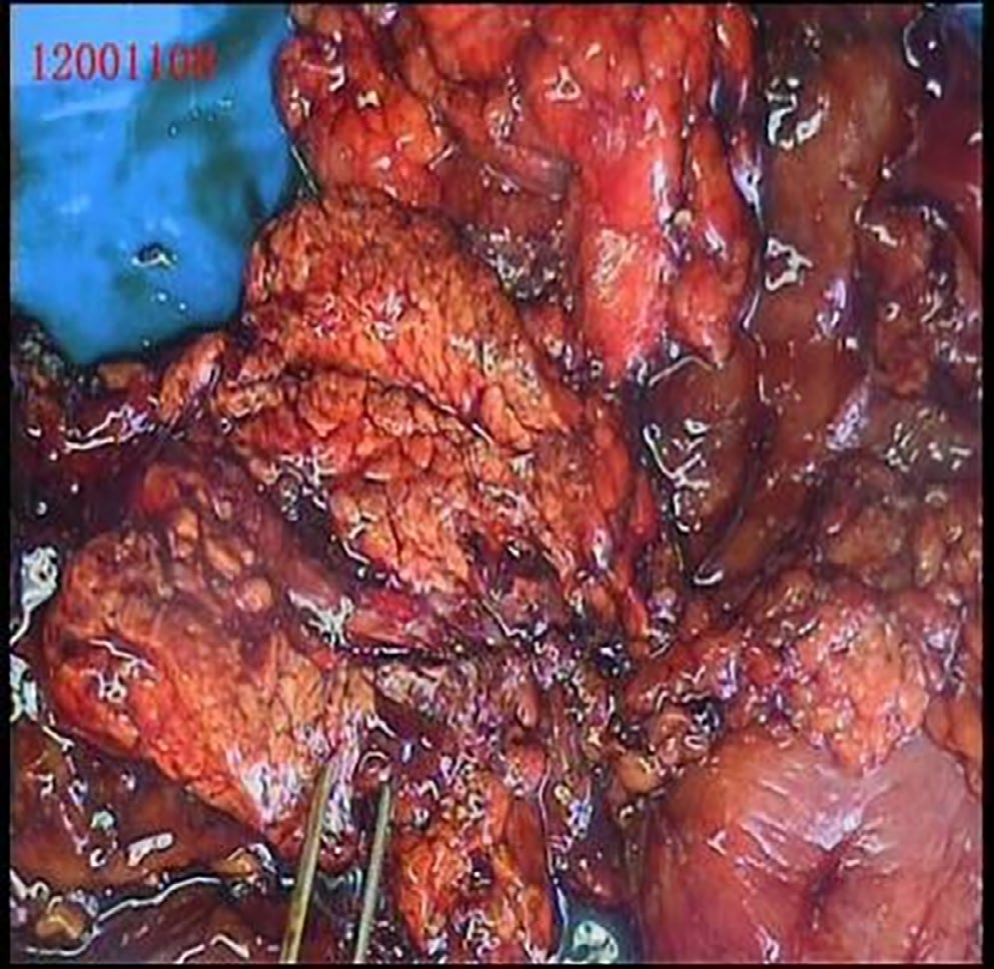
Surgical removal of tumor and pancreatic tissue.

**FIGURE 4:**
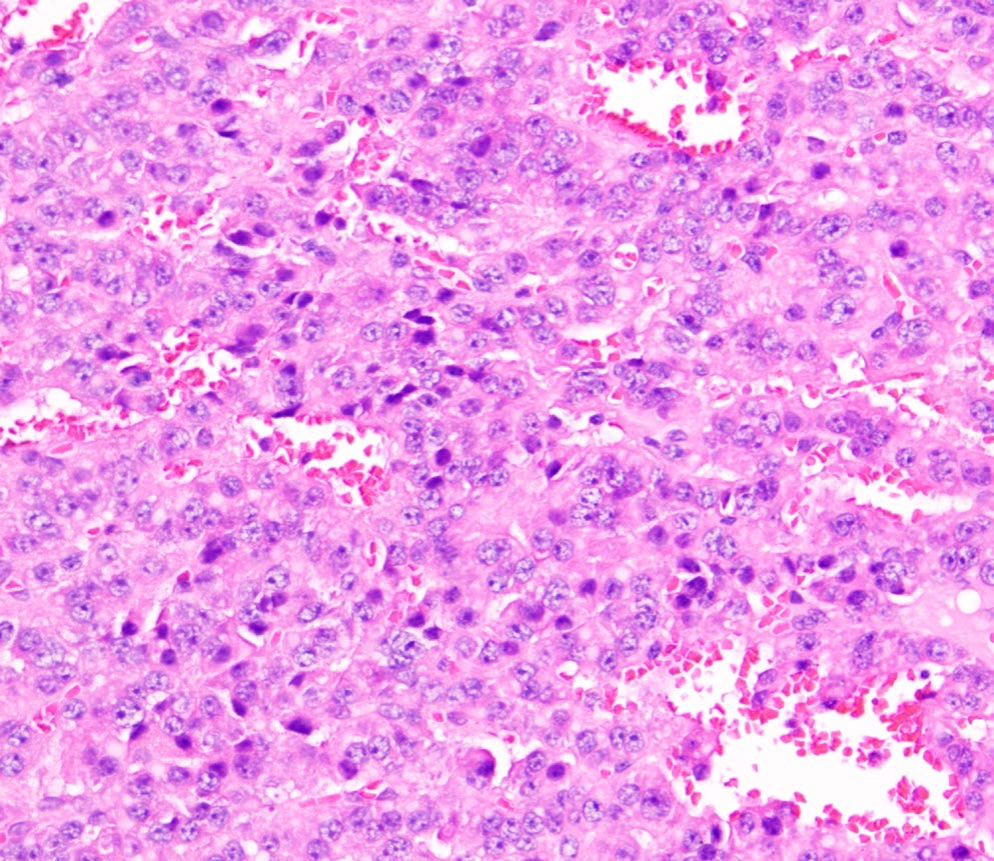
In the pancreatic tissue, there is proliferation of atypical cells, forming glandular and small nest‐like structures, with uniform tumor cells and minimal atypia.

Upon experiencing dizziness and palpitations following admission, intravenous blood glucose measured 32.6 mg/dL (1.8 mmol/L), insulin level was 108.7 μIU/L, and the insulin release index (IRI/G) was calculated as 3.3. The results of the glucose tolerance test, C‐peptide measurement, and insulin release test are presented in Table 1. Abnormal findings included blood tests, liver and kidney function, glycosylated hemoglobin levels, tumor markers, adrenocorticotropic hormone, cortisol, thyroid function, parathyroid hormone, growth hormone, and sex hormones levels were within normal limits (Tables 2 and 3). Abdominal imaging revealed no abnormalities. Ultrasound endoscopy revealed a hypoechoic nodule in the pancreatic neck, measuring approximately 11.2 × 12.7 mm (Figure 1). Ultrasound endoscopy‐guided puncture of the pancreatic nodule revealed pancreatic tissue strip lesions (Figure 2), characterized by epithelioid cells arranged in a nest‐like pattern. Uniform cell size and cytoplasmic richness were noted. A neuroendocrine tumor was suspected. Following transfer to general surgery department, post‐abdominal exploration revealed an approximately 1 cm diameter nodule in the pancreatic neck (Figure 3). Intraoperative ultrasound confirmed a hyperechoic nodule in the same location. Pancreaticoduodenectomy was subsequently performed. The postoperative autopsy specimen revealed a well‐defined mass, measuring 1.2 × 1.4 cm, located in the pancreatic neck. The intraoperative rapid pathology report confirmed the presence of a neuroendocrine tumor. Postoperative histopathology demonstrated anisotropic cell proliferation within the pancreatic tissue, characterized by glandular duct‐like and small nest‐like structures (Figure 4). The tumor cells exhibited uniformity and minimal heterogeneity, consistent with a neuroendocrine tumor.

**TABLE 1:** Glucose tolerance test, C‐peptide and insulin release tests.

	0 min	30 min	60 min	120 min	180 min	240 min	300 min
Serum glucose (mmol/L)	3.2	9.8	12.2	10.5	6.7	3.4	2.1
Insulin (μlu/mL)	49.4	108.8	147.6	76.8	46.7	41.4	53.3
C‐peptide (ng/mL)	6.7	12.6	17.1	12.5	9.1	7.5	8.1
Insulin release index	0.84	0.62	0.67	0.41	0.46	0.68	1.42

**TABLE 2:** Baseline investigations.

Variables	Results	Reference range adults
Blood		
Wbc (per μL)	1060	3500–9500
Hgb (g/dL)	131	115–150
Absolute neutrophils count (per μL)	8300	2000–7000
Lymphocyte count (per μL)	1770	1000–3000
Platelets count (per μL)	259,000	150,000–400,000
PT (s)	11.1	9.5–13.5
APTT (s)	23.6	21.0–38.0
INR	0.96	0.8–1.5
Urea (mmol/L)	3.4	1.4–9.2
Creatinine (μmol/L)	48	35–80
Na (mmol/L)	146	137–147
K (mmol/L)	3.9	3.5–5.3
Cl (mmol/L)	111	99–110
Calcium (mmol/L)	2.1	2.0–2.6
Amylase (U/L)	35	0–115
Lipase (U/L)	29	< 60
Albumin (g/L)	44	40–55
Total protien (g/L)	74	66–87
ALT (U/L)	18	< 40
AST (U/L)	12	< 31
CRP (mg/L)	1.8	0–10
HbA1c%	5.1	3.8–5.8

Abbreviations: ALT, alanine transaminase; AST, aspartate aminotransferase; Cl, chloride; CRP, C‐reactive protein; HbA1c, hemoglobin A1c; Hgb, hemoglobin; INR, international normalized ratio; K, potassium; Na, sodium; PT, prothrombin time; PTH, parathyroid hormone; PTT, partial thromboplastin time; WBC, white blood cells.

**TABLE 3:** Hormone levels.

Variables	Results	Reference range adults
PTH pmol/L	4.5	1.6–6.90
ACTH at 8 am pmol/L	3.1	1.6–13.9
Cortisol at 8 am nmol/L	209.9	133–537
PRL ng/mL	3.9	3.3–26.7
TSH uIU/mL	1.0	0.6–5.9
FT3 pg/mL	2.9	2.1–4.2
FT4 pg/mL	0.8	0.6–1.3

Abbreviations: ACTH, adrenocorticotropic hormone; FT3, free triiodothyronine; FT4, free thyroxine; PRL, prolactin; PTH, parathyroid hormone; THS, thyroid stimulating hormone.

### 3.1 Outcome and Follow‐Up

Two weeks post‐surgery, the patient exhibited elevated blood glucose levels. Fasting blood glucose measured 274.3 mg/dL (15.2 mmol/L), and 2‐h postprandial blood glucose was 334.3 mg/dL (19.1 mmol/L) (Table 4), indicating secondary diabetes mellitus. Insulin therapy was initiated for glycemic control. Following hospital discharge, the patient used insulin sporadically, without experiencing hypoglycemia. Six months post‐surgery, fasting blood glucose was 284.6 mg/dL (15.8 mmol/L), and glycosylated hemoglobin was 12.5%. The patient received instructions to use insulin consistently. Over the subsequent 4 years, no hypoglycemic episodes occurred, and glycemic control remained reasonable.

**TABLE 4:** Postoperative glucose, insulin and C‐peptide changes.

	Fasting blood glucose (mmol/L)	2‐h postprandial glucose (mmol/LS)	Fasting insulin (ulu/mL)	Fasting C‐peptide (ng/mL)
Two weeks postoperative	15.24	18.57	7.07	1.91
Six months post‐operative	15.81	—	4.76	0.2

## 4 Discussion

Insulinomas are commonly encountered as solitary benign tumors, typically measuring less than 2 cm in diameter, while malignant insulinomas are larger in size, with an average size of about 4–5 cm in diameter, and they also have higher levels of insulin and C‐peptide in the serum [5, 6]. The occurrence of benign insulinoma tumors is evenly distributed across the tail, body, and head of the pancreas [6, 7]. Insulinomas typically present with significantly elevated insulin levels. The 5‐year survival rates differ markedly between benign and malignant tumors, standing at 95.4% and 66.8%, respectively [6]. Insulinomas are the second most common neuroendocrine tumors in patients with multiple endocrine neoplasia type‐1 (MEN‐1), accounting for 10%–15% of MEN‐1 patients [8]. The age of onset is earlier than that of sporadic insulinomas, usually occurring in patients aged 10–39 [9, 10]. Despite extensive research, the etiology and pathogenesis of insulinomas remain elusive. Clinically, the patients with an insulinoma manifest primarily as episodic hypoglycemia, primarily due to excessive insulin secretion from the tumor tissue. The diagnostic criteria for insulinoma include the Whipple's triad sign: (1) spontaneous periodic episodes of hypoglycemic symptoms, coma, and associated psychoneurological symptoms, often occurring during fasting or after physical exertion; (2) blood glucose levels below 50 mg/dL (2.8 mmol/L) during these episodes; and (3) prompt resolution of the symptoms following oral or intravenous glucose administration. In cases with atypical clinical manifestations, a 72‐h starvation test is essential for a definitive diagnosis [2, 11, 12]. In addition to insulinoma causing hypoglycemia, other diseases can also lead to similar symptoms. For instance, hypothyroidism, due to the reduced secretion of thyroid hormones, can slow down the absorption of sugar in the intestines, potentially leading to fasting hypoglycemia. However, the symptoms are generally mild, primarily as general weakness, cold intolerance, dry skin, and edema. Additionally, patients with chronic adrenal cortex insufficiency are more sensitive to insulin, and most may experience hypoglycemic symptoms. These patients may exhibit distinctive pigmentation, fatigue, weight loss, and low blood pressure. Furthermore, for patients with hypoglycemia accompanied by hyperinsulinemia, it is necessary to rule out the presence of early‐stage diabetes, as the β‐cells' delayed response to glucose‐stimulated insulin secretion may lead to a significant release of insulin after meals, resulting in postprandial hypoglycemia. This can be differentiated by completing glucose tolerance and 72‐h fasting tests. For patients whose hypoglycemic episodes are primarily characterized by neurological symptoms, it is also essential to be vigilant for possible conditions such as encephalitis or subarachnoid hemorrhage. The symptoms and the test results of the patient in our reported case meet the qualitative diagnostic criteria for insulinoma. Benign insulinomas commonly respond well to surgical resection, and we now have a variety of alternative therapeutic options. These include endoscopic ultrasound‐guided radiofrequency ablation, percutaneous alcohol ablation, and trans‐arterial embolization of the insulinoma [13, 14]. The preoperative localization of insulinomas directly impacts the clearance rate, reoperation rate, and prognosis of the lesion. Accurate localization diagnosis remains a significant challenge in insulinoma diagnosis and treatment. Advancements in imaging technology have ushered in an era of simple and non‐invasive preoperative localization and diagnosis of insulinomas. Invasive techniques, such as digital subtraction angiography, percutaneous hepatic portal vein blood sampling, and selective arterial calcium‐stimulated venous blood collection, have fallen out of favor. Routine imaging modalities, including ultrasound of the upper abdomen, magnetic resonance imaging (MRI), contrast‐enhanced computed tomography (CECT) of the abdomen, and pancreatic CT perfusion imaging, are widely employed for localization and diagnosis. In a single‐center study, 286 patients with functional pNENs were included, of whom 266 had insulinomas. The study demonstrated that CT, digital subtraction angiography (DSA), contrast‐enhanced ultrasound, and MRI achieved favorable localization and diagnostic rates of 76.2%, 83.8%, 87.1%, and 92.9%, respectively [15]. Computed tomography (CT) and magnetic resonance imaging (MRI) serve as primary imaging modalities for localization and diagnosis of insulinomas. However, their sensitivity remains inadequate, particularly for small‐volume lesions. Improving the detection rate and addressing deficiencies in lesion characterization are ongoing challenges. Emerging imaging techniques, including 68Ga‐DOTA‐Tyr3‐Octreotate Positron Emission Tomography‐Computed Tomography (68Ga‐DOTATATE‐PET‐CT) and 68Ga‐NOTA‐exendin‐4 Positron Emission Tomography‐Computed Tomography (68Ga‐NOTAexendin‐4PET‐CT), and other radionuclide receptor imaging tests, exploit receptor‐binding based imaging for precise localization. The expression of various hormone receptors on the surface of neuroendocrine tumors, including insulinomas, aids in their diagnosis through the use of radiolabeled peptides. Insulinomas in particular exhibit receptors such as the growth inhibitory receptor 2 (SSTR2) and the glucagon‐like peptide‐1 receptor (GLP‐1 receptor), with over 90% of these tumors expressing the GLP‐1 receptor. The 68Ga‐NOTA‐exendin‐4 PET‐CT technique leverages the GLP‐1 receptor agonist exenatide, which is radiolabeled with 68Ga, to visualize an abnormal concentration of radioactivity in the tumor region of patients with insulinoma. Additionally, 68Ga‐DOTATATE‐PET‐CT is a growth inhibitory receptor imaging modality that employs targeted imaging with growth inhibitory analogues, such as radiolabeled octreotide, to enhance the visualization of these tumors. In a prospective study, 68Ga‐DOTATATE‐PET‐CT demonstrated superior sensitivity for lesion detection and tumor‐staging guiding, compared to whole‐body diffusion‐weighted MRI (WB DWI) and 99mTc‐HYNIC‐Octreotide SPECT/CT. The diagnostic performance of 68Ga‐DOTATATE‐PET‐CT for pancreatic neuroendocrine tumors were as follows: sensitivity 100%, specificity 80%, accuracy 84%, positive predictive value 57%, and negative predictive value 100% [16]. Despite their clinical significance, wider adoption of these novel imaging tests remains limited due to availability constraints in many hospitals. In this case, abdominal enhancement CT failed to detect the lesion, and radionuclide receptor imaging was not performed. Hence, alternative localization methods are necessary for further clarification.

Given that surgical removal of the tumor remains the sole effective treatment, to prevent undue harm to the patient resulting from misdiagnosis, a preoperative “gold standard” diagnosis is imperative, which necessitates the pathologic examination. Endoscopic ultrasound (EUS) offers the advantages of high resolution, proximity, and minimal interference from intestinal gas. During both preoperative and intraoperative phases, it allows comprehensive observation of the lesion from all angles, eliminating blind spots and facilitating precise tumor localization. In comparison to alternative imaging modalities, endoscopic ultrasound offers notable benefits, particularly in enhancing the detection rate of small lesions. Additionally, it can be used in conjunction with fine‐needle aspiration biopsy to perform histologic and cytologic examinations on the lesion. Furthermore, it enables qualitative diagnosis of the lesion, informing treatment decisions. In situations where conventional imaging results are inconclusive, it serves as a crucial complementary tool [17].

However, EUS as an invasive procedure has several drawbacks. The diagnostic effectiveness of insulinomas via EUS is constrained by the location and size of the tumors. Specifically, EUS is less effective in detecting insulinomas situated in the tail or the leptomeningeal region of the pancreas [18], both of which tend to be small. The reliability of EUS depends on the subjective interpretation of the sonographer, introducing limitations. Detecting isoechoic insulinomas poses challenges for EUS, and it cannot identify extra‐pancreatic lesions like lymph node or liver metastases. In this case, the patient underwent the EUS examination, and a small lesion in the pancreatic neck, a location prone to oversight, was identified. The fine‐needle aspiration biopsy was subsequently conducted to ascertain the lesion's nature, confirming it as a neuroendocrine tumor. The precise diagnosis of the lesion's location and nature enabled the patient to proceed with the next stage of surgical treatment.

After pancreaticoduodenectomy, the patient did not encounter hypoglycemic episodes. Hyperglycemia was attributed to secondary diabetes mellitus. Exogenous insulin treatment resulted in adequate glycemic control. Postoperatively, the patient underwent follow‐up, with no evidence of recurrence or metastasis.

The EUS‐guided fine‐needle aspiration biopsy (EUS‐FNAB) serves as a critical adjunctive technique when conventional imaging fails to precisely localize and diagnose occult insulinomas. Furthermore, novel molecular imaging technologies, including 68Ga‐DOTATATE‐PET‐CT and 68Ga‐NOTAexendin‐4PET‐CT, also offer precise and reliable localization of the tumors. These advancements enhance clinicians' diagnostic and therapeutic capabilities for occult insulinomas, minimizing misdiagnoses and enabling early intervention.

## Author Contributions

**Zhongqiu Guo:** writing – original draft. **Yanrong Chen:** writing – review and editing. **Ronghuo Liu:** validation. **Yuhua Chen:** conceptualization.

## Ethics Statement

The data for this case report were taken from the case clinical records and anonymized. Written patient consent was gained for this case report.

## Conflicts of Interest

The authors declare no conflicts of interest.

## Data Availability

The data for this case report were taken from the case clinical records and anonymized. Written patient consent was gained for this case report.
